# Study on Road Performance and Ice-Breaking Effect of Rubber Polyurethane Gel Mixture

**DOI:** 10.3390/gels11070505

**Published:** 2025-06-29

**Authors:** Yuanzhao Chen, Zhenxia Li, Tengteng Guo, Chenze Fang, Jingyu Yang, Peng Guo, Chaohui Wang, Bing Bai, Weiguang Zhang, Deqing Tang, Jiajie Feng

**Affiliations:** 1 School of Civil Engineering and Transportation, North China University of Water Resources and Electric Power, Zhengzhou 450045, China; cyz740513@ncwu.edu.cn (Y.C.); zhenxiali2009@ncwu.edu.cn (Z.L.); fangchenze@126.com (C.F.); yangjingyu@ncwu.edu.cn (J.Y.); baibing7777@ncwu.edu.cn (B.B.); tdq18738952643@163.com (D.T.); 18027459877@163.com (J.F.); 2 Henan Province Engineering Technology Research Center of Environment Friendly and High-Performance Pavement Materials, Zhengzhou 450045, China; 3 Technology Innovation Center of Henan Transport Industry of Utilization of Solid Waste Resources in Traffic Engineering, North China University of Water Resources and Electric Power, Zhengzhou 450045, China; 4 Henan Province University-Enterprise Research and Development Center for Green, Low-Carbon and High-Performance Road Materials, Zhengzhou 450045, China; 5 National and Local Joint Engineering Laboratory of Traffic Civil Engineering Materials, Chongqing Jiaotong University, Chongqing 400074, China; guopeng@cqjtu.edu.cn; 6 School of Highway, Chang’an University, Xi’an 710064, China; wchh0205@chd.edu.cn; 7 School of Transportation, Southeast University, Nanjing 210096, China; wgzhang@seu.edu.cn

**Keywords:** pavement materials, rubber particles, polyurethane gels, ice-breaking effect, road performance, ice breaking mechanism

## Abstract

Aiming at the problems of serious pavement temperature diseases, low efficiency and high loss of ice-breaking methods, high occupancy rate of waste tires and the low utilization rate and insufficient durability of rubber particles, this paper aims to improve the service level of roads and ensure the safety of winter pavements. A pavement material with high efficiency, low carbon and environmental friendliness for active snow melting and ice breaking is developed. Firstly, NaOH, NaClO and KH550 were used to optimize the treatment of rubber particles. The hydrophilic properties, surface morphology and phase composition of rubber particles before and after optimization were studied, and the optimal treatment method of rubber particles was determined. Then, the optimized rubber particles were used to replace the natural aggregate in the polyurethane gel mixture by the volume substitution method, and the optimum polyurethane gel dosages and molding and curing processes were determined. Finally, the influence law of the road performance of RPGM was compared and analyzed by means of an indoor test, and the ice-breaking effect of RPGM was explored. The results showed that the contact angles of rubber particles treated with three solutions were reduced by 22.5%, 30.2% and 36.7%, respectively. The surface energy was improved, the element types on the surface of rubber particles were reduced and the surface impurities were effectively removed. Among them, the improvement effect of the KH550 solution was the most significant. With the increase in rubber particle content from 0% to 15%, the dynamic stability of the mixture gradually increases, with a maximum increase of 23.5%. The maximum bending strain increases with the increase in its content. The residual stability increases first and then decreases with the increase in rubber particle content, and the increase ranges are 1.4%, 3.3% and 0.5%, respectively. The anti-scattering performance increases with the increase in rubber content, and an excessive amount will lead to an increase in the scattering loss rate, but it can still be maintained below 5%. The fatigue life of polyurethane gel mixtures with 0%, 5%, 10% and 15% rubber particles is 2.9 times, 3.8 times, 4.3 times and 4.0 times higher than that of the AC-13 asphalt mixture, respectively, showing excellent anti-fatigue performance. The friction coefficient of the mixture increases with an increase in the rubber particle content, which can be increased by 22.3% compared with the ordinary asphalt mixture. RPGM shows better de-icing performance than traditional asphalt mixtures, and with an increase in rubber particle content, the ice-breaking ability is effectively improved. When the thickness of the ice layer exceeds 9 mm, the ice-breaking ability of the mixture is significantly weakened. Mainly through the synergistic effect of stress coupling, thermal effect and interface failure, the bonding performance of the ice–pavement interface is weakened under the action of driving load cycle, and the ice layer is loosened, broken and peeled off, achieving efficient de-icing.

## 1. Introduction

Pavement needs to withstand the test of a complex natural environment during service, and the temperature disease caused by extreme temperature accounts for a significant proportion of all kinds of pavement diseases. These diseases will lead to pavement structure damage and performance degradation, seriously affecting the service life of the road and driving safety [1,2]. Most parts of China belong to seasonal frozen areas, and the low-temperature period in winter is long, which is often accompanied by rain and snow weather [3]. Snow and ice can easily lead to a significant reduction in road surface adhesion, causing vehicle tire slip and loss of control, resulting in casualties and traffic accidents, which seriously threaten the safety of public transportation [4,5,6]. In this regard, researchers have proposed many ways to melt ice and snow, such as spreading snow-melting agent, artificial-mechanical de-icing, and electrothermal snow-melting pavement. However, the above methods occupy traffic resources to varying degrees and reduce the level of road services. In this context, road active snow melting and de-icing technology has received extensive attention. Through the application of new materials, new ideas and new technologies, this technology gives the road surface the ability to melt ice and snow independently, which significantly improves the safety of road operation in winter. The low freezing point pavement [7,8] achieves ice melting and snow melting by improving the interfacial adhesion between the pavement and the ice and snow layer, but its snow-melting components are still dominated by chloride salts, resulting in the possibility that the salt residue in the lower part of the salt storage pavement cannot be released, which is difficult to meet the actual needs of environmental protection and practicality. As a branch of active ice and snow removal technology, energy conversion ice and snow removal pavement [9,10,11] has high snow-melting efficiency, but there are problems such as high construction cost and easy aging of materials. The construction technology of self-stress elastic pavement [12,13] is relatively simple, has better economy, avoids the negative impact of traditional ice and snow melting technology and has a certain environmental protection value. Therefore, it is necessary to study this aspect.

Under the premise of confirming the feasibility of ice melting and snow melting of rubber asphalt pavement, most of the relevant research focuses on the forming process, ice-breaking mechanism, snow-melting and ice-melting characteristics and influencing factors, mixture composition design and the performance improvement of rubber asphalt ice-melting pavement. Liang et al. [14] combined diatomite and crumb rubber particles to prepare a modified asphalt mixture. It was found that the incorporation of rubber particles and diatomite significantly improved the high-temperature stability and low-temperature crack resistance of the asphalt mixture. Chen Yuanzhao [13] proposed a prediction method of elastic modulus by establishing a multi-step, multi-phase micromechanical model. The research shows that an increase in rubber particles will reduce the elastic modulus of the mixture, and the elastic modulus of the mixture will increase significantly under low-temperature conditions, resulting in the weakening of the de-icing effect. The monitoring data of the test road by American researchers show that there is a problem of particle peeling in the practical application of rubber asphalt pavement, which leads to the road performance of the test section not being up to expectations, and the effect of ice and snow removal is limited. Li et al. [15] tested the aging performance of three typical rubber materials—ethylene propylene rubber, liquid silicone rubber and fluorine rubber—under different high- and low-temperature cycling aging conditions. The experimental results showed that repeated temperature cycling can cause fatigue failure, which may lead to a permanent increase in rubber compression deformation. Ma et al. [16] proposed a new comprehensive evaluation index (snowmelt performance coefficient; SMPC) and optimized the design parameters of the conductive rubber snowmelt bridge deck through the I-PSO algorithm. Shan et al. [17] found that the incorporation of terminal carboxylated nitrile rubber significantly improved the mechanical properties and low-temperature crack resistance of asphalt without changing the curing time. Although the high-temperature rutting resistance of asphalt itself is reduced, the high-temperature, low-temperature and water stability of porous asphalt concrete are improved. It can be seen that although rubber asphalt pavement can effectively alleviate the ice and snow problem of pavement in ice and snow weather, there are still some technical limitations. In engineering applications, rubber will age over time due to the influence of natural and artificial environments, lose elasticity, and decrease mechanical properties, resulting in cracked asphalt pavement. This causes great harm to the performance and service life of the road, and also means that the anti-icing function of asphalt pavement mixed with rubber particles will be weakened. Therefore, how to make up for the deficiency of rubber particle asphalt mixture in terms of strength and durability has become more and more difficult.

Polyurethane gels are a new type of polymer. It can be used to obtain a variety of products by adjusting the synthetic materials and preparation process. Because of its good performance, it has been widely used in various fields at home and abroad [4,18,19,20,21]. Polyurethane-modified asphalt mixture is mainly committed to improving the performance of asphalt. However, this does not completely make up for the shortcomings of the asphalt material itself. Therefore, researchers began to use it as a binder to completely replace asphalt in the development of polyurethane mixtures. Zheng et al. [22] found that the optimum molding time window for PERS is 40 to 50 min after the curing of polyurethane, balancing mechanical properties and minimizing springback. Gao et al. [23] found that the direct tensile change process of a polyurethane rubber particle mixture is divided into four stages. The mixture of polyurethane rubber particles and F2 polyurethane has high tensile properties at 18% content. Chen et al. [24,25] prepared a polyurethane mixture according to the gradation specification of an asphalt mixture. According to the research, it is found that polyurethane binder has outstanding performance in thermal conductivity, and its thermal conductivity is much higher than that of asphalt, so polyurethane gel mixtures can effectively shorten the time required for road icing in road laying applications and have excellent anti-icing and de-icing performance. Zou et al. [26] found optimal toughness at a 0.70 water–cement ratio with 6% rubber content, meeting mechanical pumping specifications while maintaining structural performance. Agavriloaie et al. [27] developed a polymer concrete with acrylate as the main component by implementing the polymerization reaction of acrylate. The results show that, compared with ordinary concrete, acrylate polymer concrete exhibits superior mechanical properties. The research results of Min Sun et al. [28] pointed out that the adhesion performance between basalt and polyurethane gels is better than that of limestone in the consideration of aggregate lithology and the selection of gradation, which has a positive effect on improving water stability. Li et al. [29] studied the physical and rheological properties of ACR/TPU composite modified asphalt, characterized the microscopic mechanism of active rubber powder and found that the choice of modifier dosage can be verified by digital fluorescence microscope. Wang et al. [30] found that the strain value of a polyurethane porous elastic pavement layer was significantly higher than that of ordinary porous asphalt pavement through indoor testing and numerical simulation. This phenomenon strongly confirmed the applicability and engineering advantages of polyurethane porous elastic pavement in urban road applications in cold regions.

In summary, polyurethane gel binder has the advantages of high viscoelasticity, strong chemical corrosion resistance and strong wear resistance, and it has broad application prospects in the field of road engineering. However, it is rare to study the application of polyurethane gels as a binder in the ice breaking of road pavement. Due to the poor adhesion between rubber particles and asphalt, rubber particles are prone to fall off under vehicle load, resulting in insufficient stability of the pavement structure, which, in turn, affects the durability and performance of the pavement. The emergence of polyurethane gels provides a new direction and idea for the lack of structural stability of rubber particle mixtures. Therefore, in this paper, polyurethane gels are used as the binder of new pavement. Through economical, simple and feasible modification methods, rubber particles with good bonding performance are developed and incorporated into a polyurethane gel mixture. By using the overall elastic deformation of pavement, the effect of active snow and ice breaking is realized, and the anti-skid performance and driving safety of pavement under cold weather conditions are improved, thus effectively improving the operation efficiency and traffic capacity of pavement. At the same time, it avoids the damage to infrastructure and environmental pollution caused by traditional de-icing methods and prolongs the service life of the road. It is of great significance to promote the development of green pavement materials and improve the service life of roads.

## 2. Results and Discussion

### 2.1. Performance Analysis of Modified Rubber Particles

#### 2.1.1. Analysis of Hydrophilic Properties of Modified Rubber Particles

The contact angle test results of the rubber particle surface under different treatment methods are shown in Table 1.

It can be seen from Table 1 that the contact angle between the surface of rubber particles and water decreased after different solution treatments; that is, the hydrophilicity of the surface of rubber particles was improved. The contact angle of the unmodified rubber particles was 125.5°, showing obvious hydrophobicity. After 30 min of treatment, the contact angle of the rubber particles showed a decreasing trend. The contact angle of the rubber particles treated with NaOH solution decreased from 125.5° to 88.6°, which was reduced by 29.6%. The contact angle of the rubber particles modified by NaClO solution decreased to 79.8°, which was reduced by 36.4%. The contact angle of rubber particles after immersion in KH550 solution was 72.3°, which was reduced by 42.4%. The surface of the rubber material changed from hydrophobic to hydrophilic, indicating that the surface properties of the rubber particles were significantly changed by the modification treatment: from the original hydrophobic state to partial hydrophilic characteristics, the surface wetting angle was significantly reduced, and the water contact performance was significantly improved. This change in surface properties is due to the synergistic effect of two aspects in the modification process: on the one hand, it effectively removes the hydrophobic impurity phase on the surface, and on the other hand, polar functional groups such as hydroxyl (-OH) and carboxyl (-COOH) were successfully introduced, which significantly improved the surface energy characteristics of the material. It can be seen that the improvement of the surface hydrophilicity of the rubber particles by the three modifiers is KH550 solution > NaClO solution > NaOH solution. After modification, the surface polarity and surface energy of the rubber particles are significantly improved, resulting in a decrease in the contact angle with the liquid and a significant improvement in the wettability. The optimization of this surface characteristic shows that the modified rubber particles have enhanced interfacial affinity, thereby improving their compatibility with the matrix material. This improvement is conducive to the full penetration of the polar adhesive at the interface and ultimately significantly improves the adhesion strength between the two-phase materials.

#### 2.1.2. Fine Microstructure Analysis of Modified Rubber Particles

**Figure 1:**
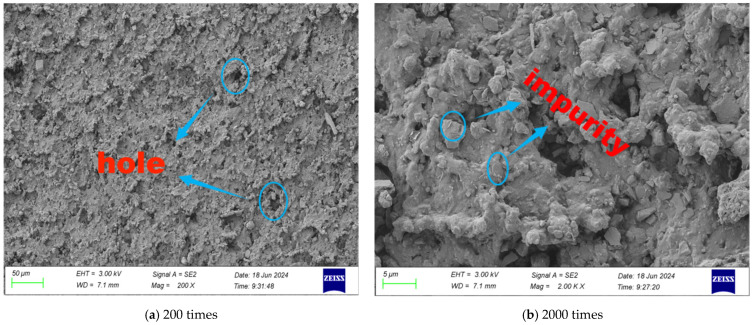
Microstructure of unmodified rubber particles.

**Figure 2:**
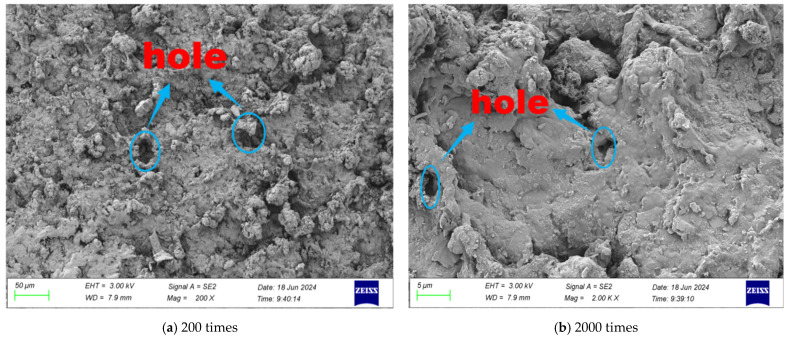
Microstructure of NaOH-modified rubber particles.

**Figure 3:**
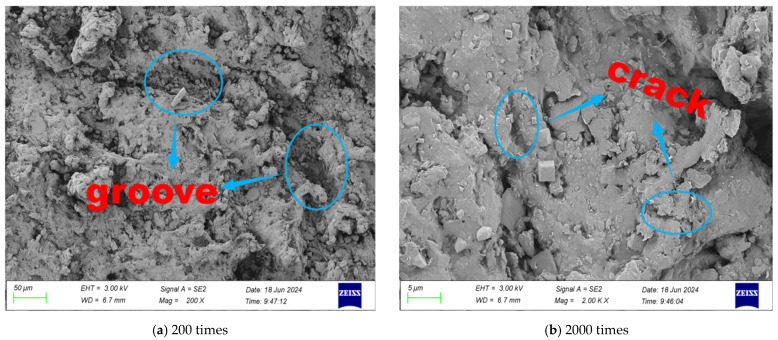
Microstructure of NaClO-modified rubber particles.

**Figure 4:**
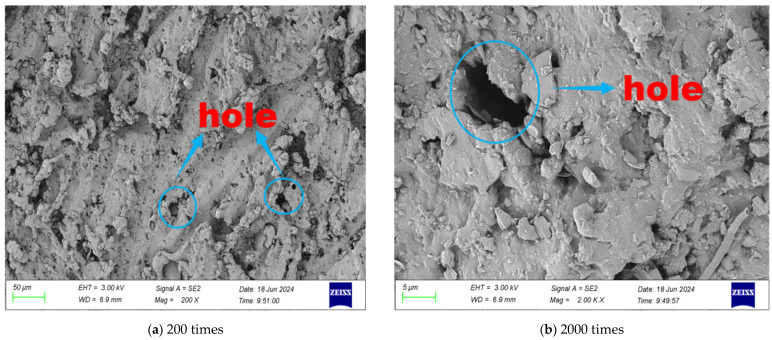
Microstructure of KH550-modified rubber particles.

SEM tests were carried out on the surface morphology of rubber particles with different modification methods, and the microscopic morphology of different multiples is shown in Figure 1, Figure 2, Figure 3 and Figure 4.

It can be seen from Figure 1 that as the magnification increases, the microstructure of the rubber particles becomes clearer and clearer. From the microscopic perspective, the surface of the rubber particles presents a rough and uneven state, and the surface is scattered with micropores. When the magnification reaches 2000 times, the bulge on the surface shows a multi-edged and sharp shape, and there is no specific regular shape. At the same time, it contains more impurity phases. These impurities are not only adsorbed dust but are also due to waste tires in the process of production and storage, as well as additives such as fatty acids and zinc oxide.

It can be seen from Figure 2 that when the magnification is 200 times, the surface microstructure of the rubber particles modified by NaOH solution changes significantly: the number of corroded micropores increases greatly, the pore size increases and the surface roughness increases significantly. At a high magnification of 2000 times, the surface impurity phase of the modified rubber particles was significantly reduced, and the surface convex structure showed round and smooth morphological characteristics. NaOH modification not only improves the porosity of rubber particles through water washing but also makes the pore structure clearer, the pore size distribution more uniform and the surface morphology more rough and uneven.

This significant change in surface morphology is mainly attributed to the removal effect of residual additives in rubber particles. The surface pretreatment with NaOH solution can effectively remove the zinc stearate coating layer on the surface of rubber particles through saponification reaction, remove the residual solvent on the surface, and form a porous structure on the surface of rubber particles. This surface modification significantly improves the roughness and porosity of the rubber particles, which is conducive to enhancing the interfacial bonding performance between the cementitious material and the rubber particle interface and improving the overall strength of the mixture.

It can be seen from Figure 3 that after NaClO solution treatment, the surface wrinkles of rubber particles increased significantly, and a large number of grooves and pores were formed, which significantly improved the unevenness of the surface. The main reason for the change in surface morphology is that NaClO solution can effectively remove the residual solvent and low molecular weight organic matter on the surface of rubber particles, thus forming a porous structure on the surface. Compared with the untreated rubber particles, the surface roughness of the modified rubber particles was significantly improved, and a large number of micron-sized voids and microcracks were generated.

According to the mechanical meshing theory, the increase in the surface roughness of the clay provides more permeation channels and anchoring points for the binder molecules. During the curing process, the adhesive can fully penetrate into the surface pores and microcracks to form a three-dimensional interlocking structure, thereby significantly improving the interfacial bonding strength. This surface modification method provides an effective technical way to improve the interfacial compatibility between rubber particles and cementitious material matrix.

After being modified by the KH550 silane coupling agent, the surface morphology of rubber particles obviously changed. Due to the chemical adsorption and self-assembly of the coupling agent molecules on the surface of the rubber particles, the surface morphology of the rubber particles develops in the direction of flattening, and the continuity of the surface protrusions is significantly enhanced. At the same time, a large number of villous protrusions were formed on the surface of rubber particles, which further improved the surface roughness. As shown in Figure 4, the surface roughness of the rubber particles modified by KH550 is higher, the porosity is larger and the surface structure is looser. The number and continuity of surface corrosion pores increase significantly, which provides more anchorage points for interface bonding. The increase in surface roughness enhances the mechanical meshing effect. The three-dimensional fluffy topology formed on the surface of rubber particles increased significantly after modification. The synergistic effect of these surface characteristics significantly enhances the interfacial bonding strength between the rubber particles and the cementitious material matrix, thereby effectively improving the working performance of the rubber particle mixture.

#### 2.1.3. Phase Composition Analysis of Modified Rubber Particles

Table 2 summarizes the results of energy spectrum composition analysis on the surface of rubber particles after different modification methods.

It can be seen from Table 2 that the types of surface elements of rubber particles treated by three modification methods are reduced, indicating that the surface impurity phase is effectively removed. Among them, the content of Zn element is significantly reduced, which confirms that the removal effect of zinc stearate is obvious. The mass fraction of Zn element on the surface of rubber particles modified by NaOH decreased by 84%, which was mainly attributed to the fact that NaOH solution could effectively dissolve the zinc stearate layer remaining on the surface of rubber particles during the crushing process of waste tires. The modification of NaClO led to a significant increase in the content of O element on the surface of rubber particles, indicating that NaClO successfully achieved surface activation and introduced more oxygen-containing functional groups, such as hydroxyl (-OH) and carboxyl (-COOH). This chemical modification changed the surface morphology of rubber particles from relatively smooth to wrinkles and grooves. In contrast, the KH550 silane coupling agent treatment formed a uniform silane layer on the surface of the rubber particles. This interface transition layer effectively filled the surface grooves and wrinkles, making the surface morphology tend to be flat. This modification not only improved the surface characteristics but also enhanced the interface compatibility between the rubber particles and the matrix material.

Compared with unmodified and NaOH- and NaClO-modified rubber particles, the mass fraction of Si element on the surface of rubber particles increased significantly after KH550 modification. This phenomenon is attributed to the chemical grafting reaction between the KH550 silane coupling agent and the surface of rubber particles. The silanol group (-SiOH) generated after the hydrolysis of KH550 undergoes a dehydration condensation reaction with the oxygen-containing functional groups (such as -OH, -COOH) on the surface of the rubber particles. At the same time, the adjacent R group forms a stable Si-O-Si covalent bond through condensation, thus constructing a uniform silane coupling agent structure layer on the surface of the rubber particles. The organic functional groups in the molecular structure form chemical bonds with the surface of the rubber particles and play a molecular bridging role at the inorganic–organic interface. This interfacial modification significantly improves the interfacial compatibility between rubber particles and cementitious materials, enhances the interfacial bonding strength and further optimizes the mechanical properties of the mixture.

### 2.2. Polyurethane Gel Dosage Test Analysis

**Figure 5:**
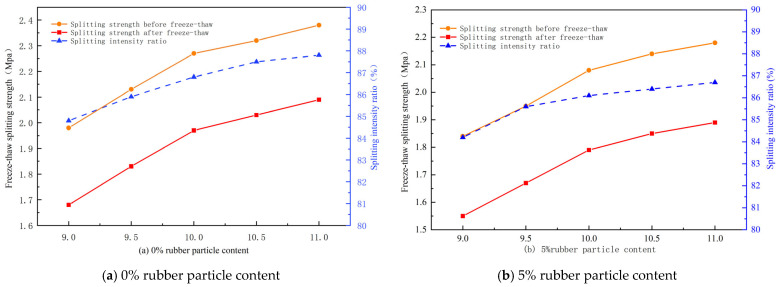

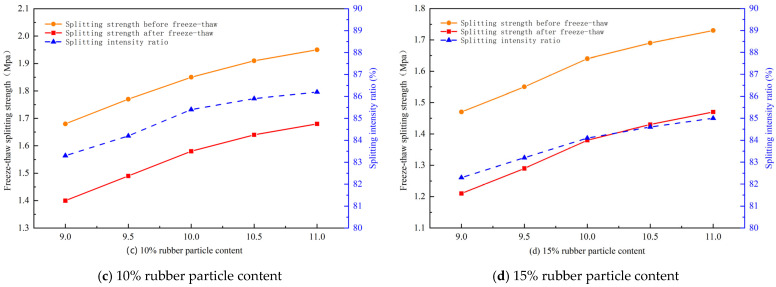
Freeze–thaw splitting strength–polyurethane gel change curve of mixtures with different rubber particle contents.

According to the relevant specifications [31], the freeze–thaw splitting test was carried out. The relationship between the splitting strength and the splitting strength ratio and the amount of polyurethane gels was shown in Figure 5.

It can be found from Figure 5 that the splitting strength ratio of the mixture prepared by replacing the fine aggregate with four kinds of rubber particles of 0%, 5%, 10% and 15% meets the technical requirements of the specification, which indicates that the polyurethane gel mixture shows good water damage resistance under different rubber particle content conditions. The freeze–thaw splitting strength of the mixture is positively correlated with the amount of polyurethane gels, but with an increase in the amount, the strength growth shows obvious stage characteristics. Under the same rubber particle content, when the amount of polyurethane gels increased from 9–9.5% and then to 10%, the freeze–thaw splitting strength of the specimen showed a rapid upward trend; however, when the amount of polyurethane gels continued to increase from 10% to 10.5% and then to 11%, the strength growth rate significantly slowed down. Taking RPGM mixed with 10% rubber particles as an example, when the amount of polyurethane gels increased from 9–9.5% and then to 10%, the freeze–thaw splitting strength ratio of the specimens increased by 1.1% and 1.4%, respectively; while the amount increased from 10–10.5%, the strength only increased by 0.5%. This is because the strength of the mixture mainly depends on the bonding force formed by the polyurethane gel binder at the aggregate interface. Therefore, increasing the amount of glue at the initial stage can significantly improve the interface bonding effect. However, due to the limited contact surface of rubber particles in the mixture, the excessive amount of glue only leads to the thickening of the adhesive film on the aggregate surface, which has a limited effect on the overall strength improvement, thus making the strength growth trend gradually flatten out.

In summary, when the amount of polyurethane gels increases to 10%, the strength of the RPGM increases most significantly, and then the growth rate gradually slows down, and the water damage resistance of the mixture fully meets the use requirements. From the perspective of engineering economy and performance, in order to ensure that the mixture has sufficient water damage resistance reserve and to avoid cost waste caused by excessive use, the optimal polyurethane gel dosage of the mixture is determined to be 10%.

### 2.3. Road Performance Analysis of Mixture

#### 2.3.1. Elevated Temperature Property

**Figure 6:**
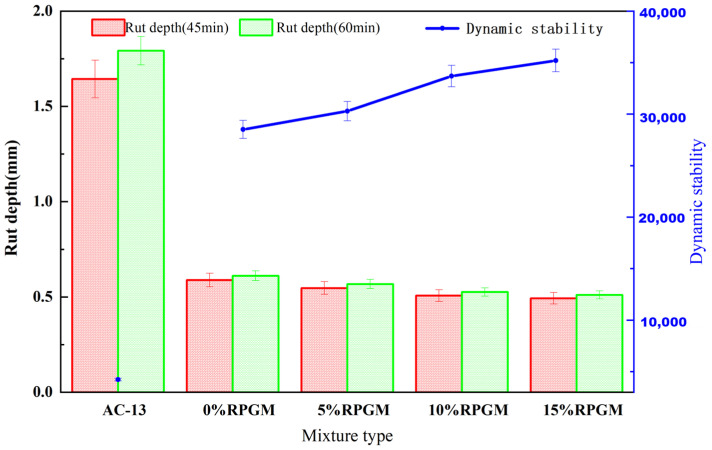
Rutting deformation and dynamic stability change curve.

As the evaluation index of the rutting test indicates, dynamic stability is positively correlated with the high-temperature performance of the mixture. The results after three tests are shown in Figure 6.

It can be seen from Figure 6 that the deformation of RPGM is significantly lower than that of the ordinary asphalt mixture. The dynamic stability of the AC-13 asphalt mixture is 4229 times/mm, and the dynamic stability of RPGM with 0% rubber particle content is 28,507 times/mm, which is more than 7 times that of the former, and shows an upward trend with an increase in rubber particle content. It can be seen that RPGM has an absolute advantage in terms of high-temperature stability. This performance advantage is due to the essential difference between the two binders: asphalt binder is highly sensitive to temperature, and it is easy to soften and flow under high-temperature conditions, resulting in insufficient high-temperature stability of the asphalt mixture. Polyurethane gel binder is a kind of thermosetting material, and its two components form cross-linking solidification through a chemical reaction, resulting in excellent thermal stability, so that the polyurethane gel mixture has excellent high-temperature rutting resistance.

Compared with RPGM without rubber particle content, the dynamic stability of RPGM with 5% rubber particle content increased by 6.2%. The dynamic stability of RPGM with 10% dosage increased by 18.2%. The dynamic stability of RPGM with 15% rubber particle content increased by 23.5%. This shows that rubber particles have a significant effect on enhancing the anti-rutting performance of the mixture. This is because the rubber particles have a low elastic modulus, and the external load is converted into internal elastic potential energy through elastic deformation. Under the action of wheel load, the rubber particles undergo recoverable deformation, thus effectively dispersing the stress concentration and reducing the strain concentration of the mixture. Due to the excellent elastic recovery performance of rubber particles, they can be quickly restored to the original state after unloading, thereby reducing the permanent deformation of the mixture.

#### 2.3.2. Low-Temperature Performance

**Figure 7:**
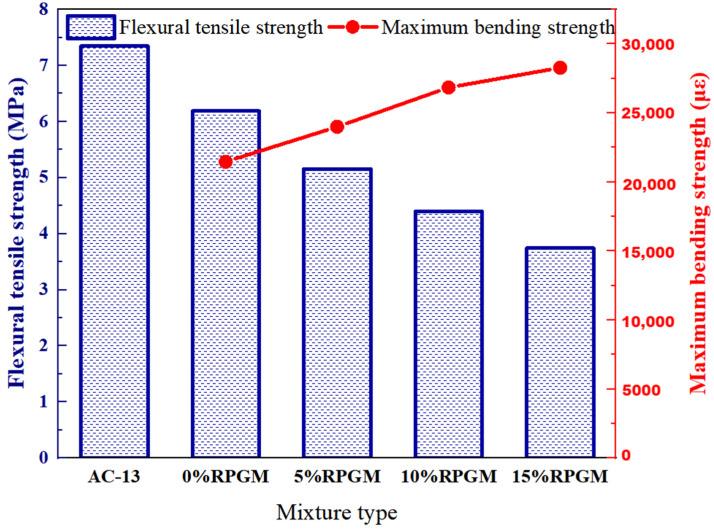
Low-temperature bending test results.

The maximum flexural tensile strain at the bottom of the beam was selected to characterize the low-temperature performance of the rubber polyurethane gel mixtures. The results after four tests are shown in Figure 7.

It can be seen from Figure 7 that the addition of rubber particles significantly increased the maximum bending strain of RPGM. When the content was 5%, 10% and 15%, the maximum bending strain was 11.8%, 25.1% and 31.7% higher than that of RPGM with 0% rubber particles. However, the incorporation of rubber particles has a negative impact on the flexural tensile strength. The flexural tensile strength of RPGM with 5%, 10% and 15% rubber particles decreased by 16.6%, 28.9% and 39.6%, respectively, compared with RPGM without rubber particles. This is mainly due to the introduction of rubber particles, which reduces the overall strength of the mixture and makes the material more prone to cracking when stressed.

Although the flexural strength of RPGM is slightly lower than that of the traditional asphalt mixture, its maximum flexural strain value is much higher than that of the latter, which is more than 7 times that of the asphalt mixture. With an increase in rubber particle content, the elastic deformation ability of the mixture is significantly enhanced, which further verifies that RPGM has good deformation ability, so that it can effectively absorb and disperse stress under complex load conditions, thereby improving the durability of the pavement. It can be seen that the introduction of rubber particles improves the low-temperature performance of the mixture, mainly due to its excellent stress relaxation characteristics, so that RPGM can still maintain good deformation adaptability when the temperature drops sharply, inhibiting the generation of low-temperature shrinkage cracks and prolonging the service life of the pavement.

#### 2.3.3. Water Stability

**Figure 8:**
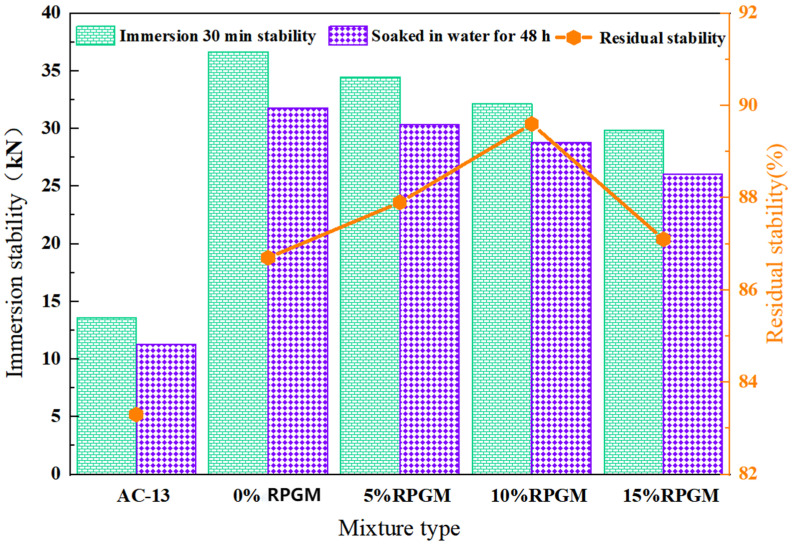
Results of Marshall immersion test.

**Figure 9:**
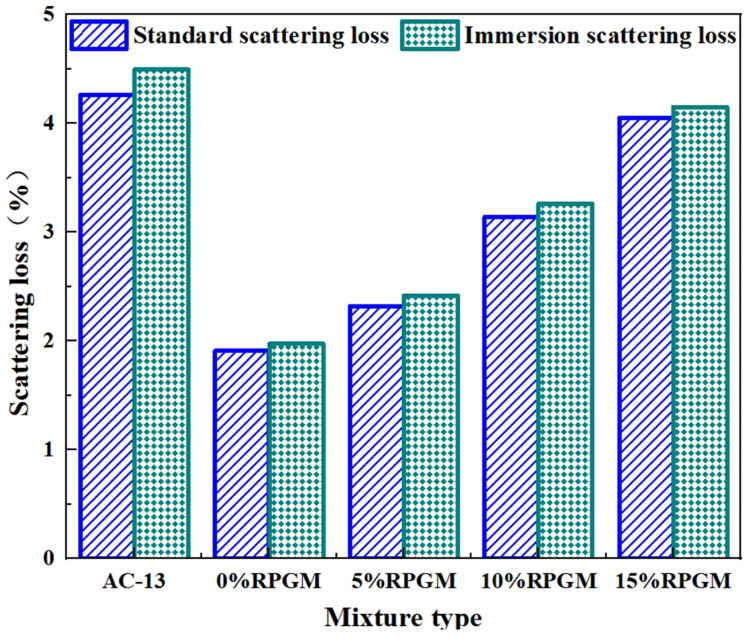
Results of immersion scattering test.

The water stability of RPGM was evaluated by the immersion Marshall test and the immersion dispersion test. The test results are shown in Figure 8 and Figure 9.

It can be seen from Figure 8 that the residual stability of RPGM is much higher than that of the AC-13 asphalt mixture under four kinds of rubber particle substitution, indicating that RPGM has better water damage resistance under different rubber particle contents. Moreover, with the increase in rubber particle content, the residual stability of RPGM showed a trend of increasing first and then decreasing. Compared with the polyurethane gel mixture without rubber particles, the residual stability of RPGM increased by 1.4%, 3.3% and 0.5%, respectively, indicating that increasing the content of rubber particles can improve the water stability of the mixture. This is due to the fact that the polarity of the optimized rubber particles and polyurethane gel molecules is relatively close, which strengthens the bonding force between polyurethane gels and rubber particles. The optimized rubber particles can fully fill the voids in the mixture, improve the compactness, reduce water intrusion and form a more stable and water-resistant structure. The higher the rubber particle content, the better the water stability of the mixture. However, when the content of rubber particles increases from 10–15%, the residual stability of the mixture shows a decreasing trend, but it is still higher than that of the ordinary asphalt mixture. This is because too many rubber particles may lead to insufficient encapsulation of polyurethane gel binder, uneven dispersion, increased porosity and other problems, affecting its bearing capacity and stability. Therefore, with the increase in rubber particle substitution, the residual stability of the polyurethane gel mixture increases first and then decreases.

Compared with the AC-13 asphalt mixture, the scattering loss rate of the mixtures with four kinds of rubber particle content decreased by 56.2%, 46.3%, 27.5% and 7.7%, showing stronger wear resistance, and the scattering resistance of the specimen was improved. In addition, the mass loss of RPGM before and after soaking is less than 5%, and it does not have a great impact with the change in rubber particle content. This shows that under the action of water, the polyurethane gel binder and aggregate still maintain a good bond strength and have good water stability. However, when the content of rubber particles continues to increase, the scattering loss rate will show a continuous upward trend. The scattering loss rate of the mixture with 5%, 10% and 15% rubber particles is 22.6%, 65.4% and 110.6% higher than that of the mixture with 0% rubber particles, respectively. This is because the bonding performance between rubber particles and polyurethane gels is worse than that of stone, which leads to the formation of a large number of ‘rubber–rubber’ contact points in the aggregate when the content of rubber particles is too high. Under the action of collision, these contact points easily lead to the separation of the aggregate, thus reducing the overall strength of the specimen and ultimately leading to an increase in the scattering loss, but it still meets the requirements. This shows that the urethane adhesive can still maintain good bonding strength under soaking conditions, and the aggregate does not easily fall off the surface of the specimen, which ensures the overall structural stability and high strength of the mixture.

#### 2.3.4. Fatigue Property

**Figure 10:**
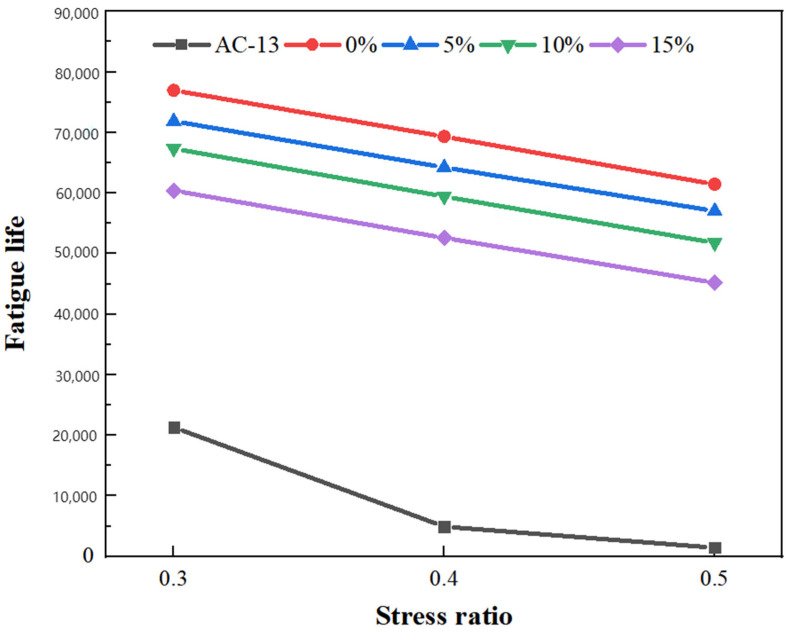
Fatigue life curve of RPGM.

The fatigue test results of RPGM are shown in Figure 10.

According to Figure 10, the fatigue resistance of the polyurethane gel mixture is much higher than that of the ordinary asphalt mixture. When the stress ratio is 0.3, the fatigue life of polyurethane gel mixtures with 0%, 5%, 10% and 15% rubber particles is 3.6 times, 3.4 times, 3.2 times and 2.8 times that of the AC-13 asphalt mixture, respectively. This is because polyurethane gels have excellent bonding performance and high strength, which can firmly bond rubber particles together to form a network structure with certain flexibility and strength. This structure makes the RPGM bear the load. Through the synergistic effect of the elastic deformation of rubber particles and the polyurethane gel network, the stress can be effectively dispersed and transmitted, and the local stress concentration can be reduced, thereby improving the fatigue resistance of the material.

When the stress level is the same and the content is different, the fatigue life of the mixture gradually decreases with the increase in the content of rubber particles, indicating that the incorporation of rubber particles reduces the fatigue performance of the polyurethane gel mixture. Under the 0.3 stress ratio, compared with the polyurethane gel mixture with 0% rubber particle content, the fatigue life of the mixture with 5%, 10% and 15% rubber particle content decreased by 6.6%, 12.5% and 21.5%, respectively. When the content is the same and the stress ratio is different, the fatigue life of the RPGM gradually decreases with the increase in the stress ratio. Taking the content of 10% as an example, the logarithmic fatigue life of RPGM at 0.4 and 0.5 stress ratios was 4.7741 and 4.7144, respectively, and the logarithmic fatigue life at 0.3 stress ratio decreased by 4.17%, 7.03% and 8.18%, respectively. It can be seen that rubber particles have a certain negative effect on the fatigue performance of the polyurethane gel mixture, and the degree of influence gradually increases with the increase in content. This is because the modulus of rubber is lower than that of polyurethane gels. Excessive incorporation will reduce the overall stiffness of the mixture, resulting in increased strain amplitude and accelerated fatigue damage.

**Figure 11:**
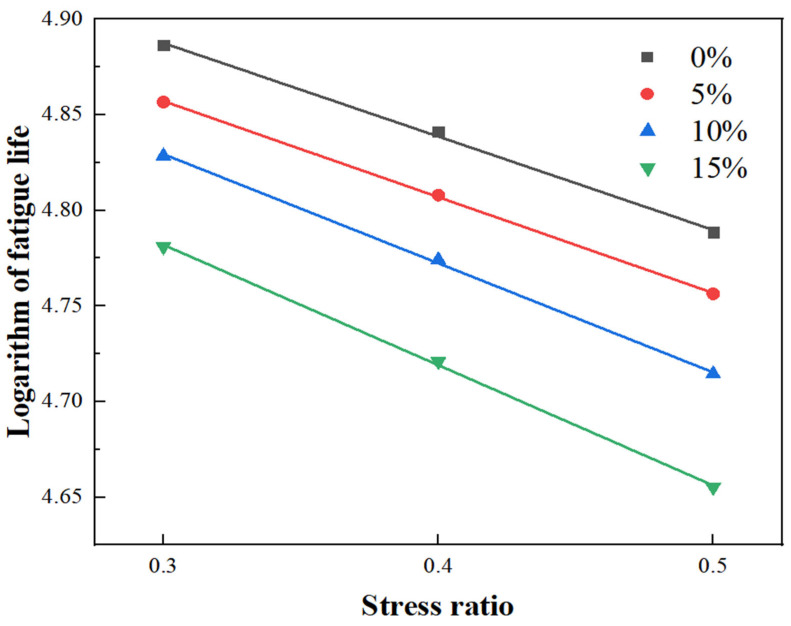
RPGM logarithmic fatigue life curve fitting results.

According to Formula (1), the fatigue life of RPGM under different stress levels is analyzed by regression fitting, and the results are shown in Figure 11.

(1) $$\log N_{f}=C-D\sigma$$

where *N_f_* is the number of load action. σ is the stress level, and *C* and *D* are characteristic parameters of fatigue test.

It can be seen from Figure 11 that after the logarithm of fatigue life is linearly fitted, the fatigue life of RPGM with different contents shows good linear regression characteristics at a 0.3~0.5 stress ratio. The correlation coefficient R^2^ obtained after fitting reached more than 99.0%, indicating that the linear equation can effectively fit the test data, so the equation can be used for the regression analysis of RPGM. Among them, the coefficient D value represents the sensitivity of the fatigue life of the RPGM to the stress level. The larger the D value, the higher the sensitivity of the fatigue life to the stress level. The C value represents the intercept of the fatigue curve on the coordinate axis. The larger the C value, the better the fatigue resistance of the RPGM. The C value decreases with the increase in rubber particle content, indicating that the fatigue performance of polyurethane gel mixtures becomes worse with the increase in rubber particle content.

#### 2.3.5. Slip Resistance

**Figure 12:**
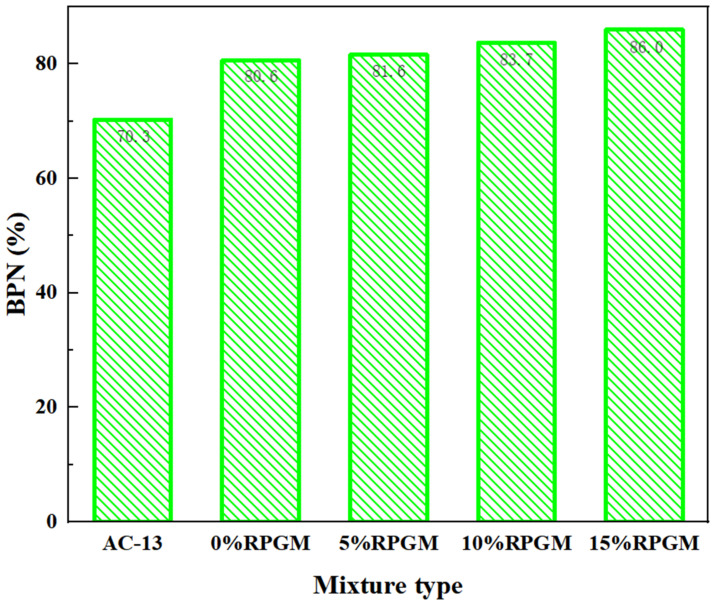
Friction coefficient test results.

Research on the anti-skid properties of icy and snowy road surfaces is of great significance for ensuring traffic safety, economically efficient winter maintenance and energy conservation. Skid resistance is a key factor in ensuring vehicle safety on road surfaces, and excellent skid resistance is a prerequisite for safe vehicle operation. This chapter follows the method outlined in the “Field Testing Procedures for Highway Subgrade and Pavement” [31] (JTG 3450-2019), recording test temperatures and measured swing values and applying temperature corrections according to Equation (2). The test results are presented in Table 3 and Figure 12.

(2) $$BPN_{20}=BPN_{T}+\Delta BPN$$

In the formula: *BPN_20_*—Converted to the pendulum value at a standard temperature of 20 °C;

*BPN_T_*—Swing value at road surface temperature T;

*ΔBPN*—temperature correction value; see Table 3.

It can be seen from Table 4 and Figure 12 that with an increase in rubber particle content, the pendulum value of RPGM continues to increase and is greater than that of the asphalt mixture. Compared with the AC-13 asphalt mixture, the BPN value increased by 14.7 units, 16.1 units, 19.1 units and 22.3 units, respectively. Note that BPN is a relative index reflecting surface friction characteristics and does not linearly correlate with the actual friction coefficient when exceeding 70. Compared with the RPGM with 0% rubber particles, the friction coefficients of RPGM with different rubber particles increased by 1.2%, 3.8% and 6.7%, respectively. This is because the rubber particles themselves have a high friction coefficient and a small modulus. When it is combined with a polyurethane gel mixture, it can improve the friction performance of the mixture surface, thereby improving the surface friction coefficient of the mixture. In addition, the optimized rubber particles have better adhesion performance with the polyurethane gel binder. After the mixture is fully mixed, the rubber particles are evenly distributed on the surface of the rutting plate of the RPGM. The surface of the rubber particles is rough, the wear resistance is good and it has good elasticity and toughness. When impacted by the pendulum, it can effectively absorb the impact energy and can quickly recover the deformation and maintain its friction coefficient. Therefore, the more rubber particles are added, the greater the friction coefficient of the RPGM and the higher the skid resistance.

### 2.4. Analysis of Ice-Breaking Effect and Mechanism of Mixture

#### 2.4.1. Phenomenon Analysis of Ice-Breaking Test

**Figure 13:**
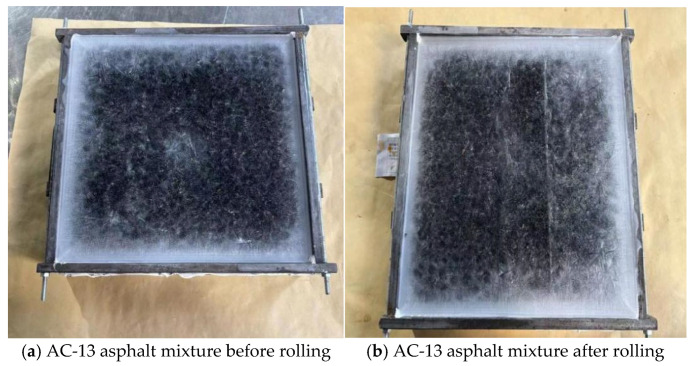
Ice-breaking rutting test.

**Figure 14:**
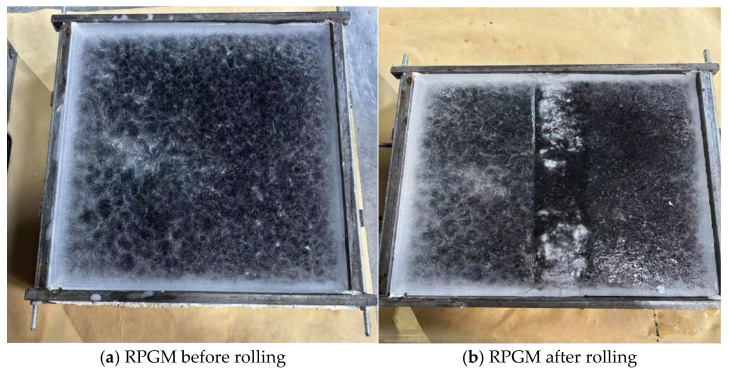
Ice-breaking rutting test.

The test was carried out at room temperature in January, during the winter in Zhengzhou City. The temperature was about 0 °C. The thickness of the ice layer of the specimen was controlled to be 4 mm. After 20 min of rolling, the phenomenon before and after the ice-breaking test of different rutting specimens is shown in Figure 13 and Figure 14.

As shown in Figure 13 and Figure 14, during the test, the rutting test piece was rolled for 20 min. The ordinary AC-13 asphalt mixture test piece did not change significantly under the reciprocating motion of the wheel, but it formed a wheel track belt. There were some fine cracks, and the ice layer did not fall off. For the rubber particle polyurethane gel mixture specimen, cracks appear on the ice surface. The ice layer at both ends of the wheel track is first broken, and then a large crack is formed. With the increase in the number of round trips of the wheel, the cracks continue to expand, and the ice layer gradually falls off, exposing the RPGM specimen. The wheel changes direction at both ends of the wheel track while applying a large tangential force to the ice layer. Due to the high elasticity of rubber particles and polyurethane gel binders, under the wheel load, the rubber particles undergo elastic deformation, while the surrounding stone deformation is small, resulting in stress concentration in the edge area of the rubber particles. This stress concentration phenomenon causes cracks in the ice and snow layer under shear failure. As the load continues to be applied, the ice and snow layer gradually forms large cracks, which eventually leads to the shedding of the ice and snow layer. In addition, during the test, it was found that the broken ice layer was more likely to fall off the rutting surface of the RPGM. This is due to the fact that the surface of the rutting plate is covered with a layer of polyurethane gel film. Polyurethane gels have the characteristics of low surface energy and high flexibility, resulting in a low interface adhesion with ice, thereby promoting the ice layer from the surface. In summary, the rubber particle polyurethane gel mixture shows better ice and snow removal performance than the traditional asphalt mixture.

#### 2.4.2. The Effect of Rubber Particle Content on Ice-Breaking Effect

**Figure 15:**
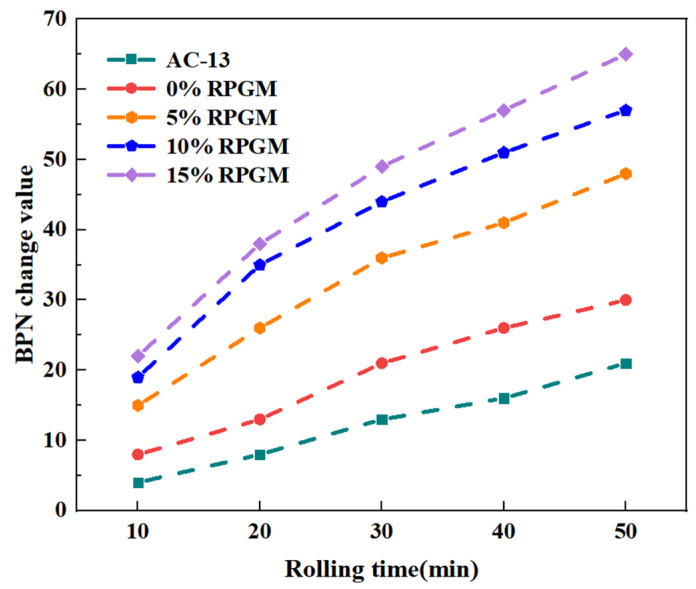
BPN change value of different mixture specimens.

In this study, 10% polyurethane gel was selected to form the rut board specimen, and the thickness of the ice layer was initially controlled at 3 mm. Through the indoor icebreaking test of RPGM with different rubber particle contents, the BPN change values were measured, respectively, and the results are shown in Figure 15.

The test results show that with the extension of rolling time, the BPN variation of the rut plate specimens shows a positive correlation trend. Compared with the AC-13 asphalt mixture specimens, the BPN variation value of RPGM increases significantly. When the rolling time is 30 min, the polyurethane gel mixture with rubber particle content of 0%, 5%, 10% and 15% increases the BPN variation value by 0.6 times, 1.8 times, 2.4 times and 2.8 times, respectively, which indicates that the ice-breaking effect of RPGM is obviously better than that of the traditional asphalt mixture. It should be noted that the growth rate of the BPN change value of the AC-13 asphalt mixture specimen will increase with the increase in rolling time. The reason for this phenomenon is that the test temperature is higher than the melting point of ice. As the rolling time continues to extend, the strength of the ice itself and the bond strength between it and the rut board decrease.

In addition, for the mixture with 0% rubber particle content, with the continuous increase in rubber particle content, the BPN change value of the specimen increases greatly. When the rolling time is 30 min, the BPN change value of the polyurethane gel mixture with 5%, 10% and 15% rubber particle content increases by 71.4%, 109.5% and 133.3%, respectively, which indicates that the introduction of rubber particles has a significant improvement effect on the ice-breaking performance of the mixture.

#### 2.4.3. The Influence of Ice Thickness on Ice-Breaking Effect

**Figure 16:**
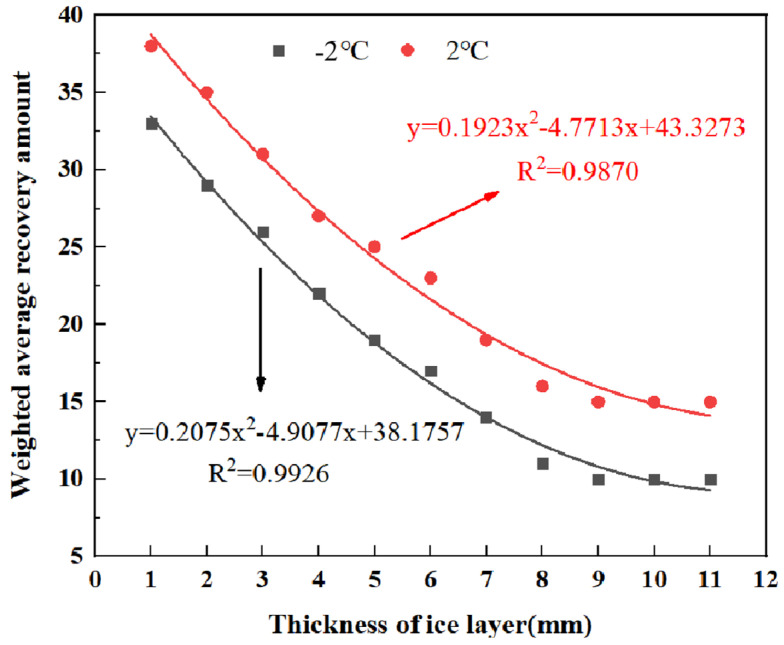
The change in pendulum weighted average recovery of specimens with different ice thicknesses.

**Figure 17:**
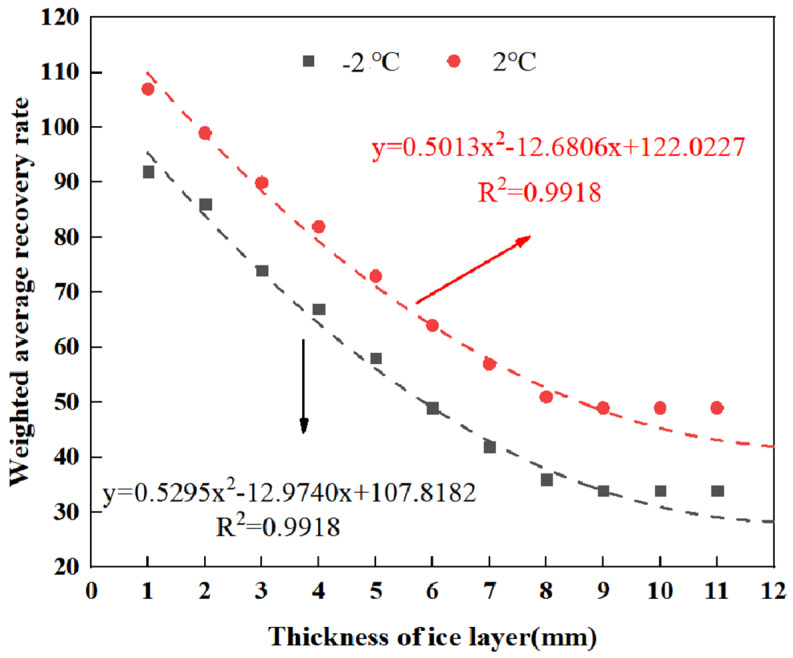
The change in swing value recovery rate of specimens with different ice thicknesses.

In order to analyze the effect of different ice thicknesses on the ice-breaking efficiency of RPGM, two indexes of weighted average recovery and recovery rate of pendulum value (as shown in Formulas (3) and (4)) were introduced. The rutting plate specimens were formed with 10% polyurethane gel content and 15% rubber particle content. The ice thickness was set to 3 mm, and the test temperature was set to −2 °C and 2 °C, respectively. The weighted average recovery and recovery rate of pendulum value at 20 min were used to characterize the de-icing effect of the mixture. The results of specimens with different ice thicknesses are shown in Figure 16 and Figure 17.

(3) $$\bar{F}=\frac{\sum \left(N,\Delta D\right)}{\sum N}$$

(4) $$\bar{\rho }=\frac{\sum \left[\left(N,\Delta D/D0\right)\right]}{\sum N}$$

where $\bar{F}$ is the weighted average rise of the pendulum value, $\bar{\rho }$ is the swing value recovery rate, N is the number of rolling, ∆D is the variation of the pendulum value and *D*_0_ is the initial pendulum value.

The results show that when the test temperature is set to −2 °C and 2 °C, the average weighted recovery of the pendulum value measured in the ice-breaking test and the corresponding recovery rate show a very consistent change trend. The correlation coefficient R^2^ obtained after fitting is more than 98%, which fully shows that there is a very significant correlation between the influence of ice thickness on the average weighted recovery amount and the recovery rate of pendulum value. From Figure 16 to Figure 17, it can be found that with the increase in ice thickness, the weighted average recovery and the average weighted recovery rate of pendulum value both show a downward trend. This downward trend is particularly significant when the ice thickness is thinner (1–6 mm). At −2 °C, the average weighted recovery changes by 12.1%, 10.3%, 13.6% and 10.5%, respectively, with the increase in ice thickness. However, with the further increase in ice thickness, the decline rate gradually slows down. When the ice thickness reaches 9 mm, the weighted average recovery amount and the pendulum average weighted recovery rate basically tend to be stable, indicating that the ice-breaking ability of RPGM has been significantly weakened, almost losing the ice-breaking function.

#### 2.4.4. Analysis of Ice-Breaking Mechanism

**Figure 18:**
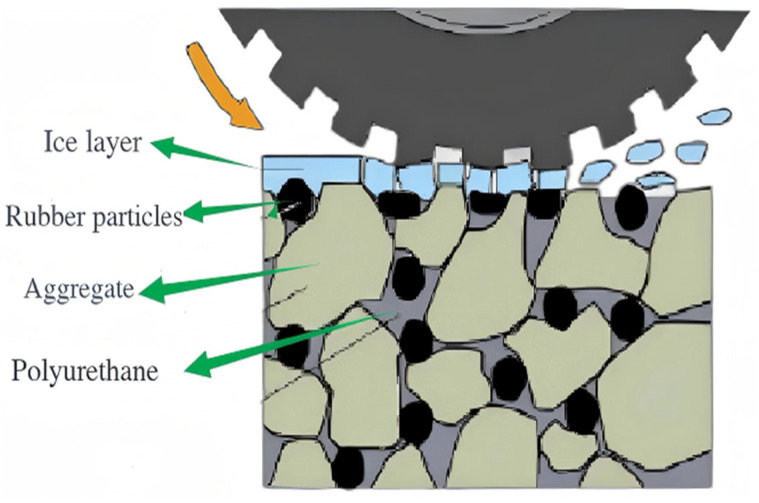
Ice-breaking mechanism of RPGM.

Ice is a dense crystal structure formed by water molecules at low temperatures. It has the characteristics of high hardness and strong adhesion, and it is not easy to break. The elastic modulus of ice varies with temperature, typically decreasing significantly with increasing temperature. Compared with snow, ice has a higher density and a closer structure, and an extremely thin quasi-liquid water film will be formed on the surface near the melting point. This layer of water film further reduces the adhesion between ice and the road surface but also makes it more difficult to remove the ice layer by conventional mechanical methods. As a new type of pavement ice-breaking material, the ice-breaking mechanism of RPGM mainly plays a role through the triple mechanism of thermal effect, stress effect and interface failure (Figure 18).

As a polymer elastomer, rubber particles will generate internal friction heat during the repeated compression and rebound process of driving load. Due to the poor thermal conductivity of rubber, the heat does not dissipate quickly, resulting in a gradual increase in the surface temperature of the particles. When the temperature is close to the melting point of ice, the local micro-area of the contact surface between ice and the mixture will melt, forming a layer of lubricating water film and weakening the bonding strength between ice and pavement. The adhesion between ice and the pavement interface is greatly reduced, and the ice layer falls off the pavement. At the same time, the RPGM has good elasticity and flexibility, which can effectively improve the deformation capacity of the road surface and change the mechanical response characteristics of the ice layer on the road surface. When the vehicle load is applied to the road surface, a large displacement will be generated, thereby changing the stress distribution of the ice layer on the road surface. Through the stress action, the bonding force between the ice layer and the road surface is destroyed. When the stress or strain of the ice layer reaches the extreme value of the damage, the ice layer reaches rupture. In addition, the rough surface of the rubber particles in the mixture forms a non-uniform contact with the ice layer, and the local stress concentration will promote the brittle fracture of the ice body. The microporous structure after polyurethane gel curing can absorb part of the ice-melting water and reduce the risk of secondary icing. It can be seen that the RPGM, by virtue of its material properties, makes the ice layer gradually loosen, break and eventually separate from the road surface, so as to achieve active de-icing. This process does not need to rely on salting or manual removal, which significantly improves the safety and traffic efficiency of winter roads while reducing maintenance costs, and it has significant environmental and economic value.

From the perspective of mechanical response characteristics, the ice layer is mainly compressed and sheared in the rectangular area below the tire, and the maximum shear stress concentration appears at the edge of the contact area, while there is a significant tensile stress concentration in the area near the rim of the double-wheel gap. Therefore, it is considered that the failure of the ice layer here is mainly caused by the instability and expansion of the composite crack (the combination of type I crack and type II crack in fracture mechanics). For the mixed fracture failure of brittle materials, the strain energy density factor theory is a widely used fracture mechanics material failure criterion. Therefore, this criterion is used to analyze the mixed fracture failure of the rubber polyurethane gel pavement ice layer.

According to the strain energy density factor criterion formula, when the polar coordinate θ = θ_0_ (θ_0_ is the cracking angle), the minimum strain energy density factor is S_min_ = Sc. The equivalent discriminant function is

(5) $$f(K_{I},K_{\mathrm{II}})=\frac{S_{\min}}{Sc}-1$$

where *S_C_* is the critical value of strain energy density factor, mm, $K_{I},K_{II}$ are the Mode and Mode Critical Stress Intensity Factors of Mixed Mode Crack Instability Propagation and *S_C_* can be determined by the fracture strength test of ice material.

(6) $$Sc=\frac{1-2v}{4\pi \mu }K_{ΙC}^{2}$$

where v is the Poisson ratio, μ is the ice shear modulus and *K*_IC_ is the mode I fracture toughness of ice material.

(7) $$\frac{\partial S}{\partial \theta }=0,\;\frac{\partial^{2}S}{\partial \theta^{2}}$$

(8) $$\theta 0=\arccos(\frac{1-2v}{3})$$

When *v* is 0.3, *θ_0_* is about −83°. Thus, the specific expression of the discriminant function is

(9) $$f(K_{I},K_{\mathrm{II}})=\frac{S_{\min}}{Sc}-1={\left(\frac{K_{I}}{K_{IC}}\right)}^{2}+0.06617\left(\frac{K_{I}}{K_{IC}}\right)\left(\frac{K_{\mathrm{II}}}{K_{IC}}\right)+1.0917{\left(\frac{K_{\mathrm{II}}}{K_{IC}}\right)}^{2}-1$$

According to the strain energy density factor theory, when the discriminant function *f*(*K*_I_, *K*_II_) < 0, that is, *Smin* < SC, the mixed mode I and II cracks will not expand; when the discriminant function *f*(*K*_I_, *K*_II_) = 0, that is, *Smin* = *SC*, the mixed mode I and II cracks begin to destabilize. When the discriminant function *f*(*K*_I_, *K*_II_) > 0, that is, S_min_ > S_C_, the mixed mode I and II cracks have become unstable. Therefore, according to the relationship between the discriminant function *f(K_I_, K_II_)* and 0, it can be judged whether the pavement ice layer will break. The fracture toughness K_IC_ of the mode I (open) fracture mode is about 80 kPa·m^1/2^. When the temperature is 0 °C and the thickness of the ice layer is 3 mm, the elastic modulus of ice is about 3000 MPa. Assuming that the elastic modulus of RPGM is 1000 MPa, it can be calculated that

(10) $$K_{I}{/K}_{\mathrm{IC}}=0{.1956671387,\;K}_{\mathrm{II}}{/K}_{\mathrm{IC}}=0.8887343329\;$$

It can be seen that *K_II_* is much larger than *K_I_*, and the fracture mode is mainly type II (hear-induced). By substituting the above values into the discriminant function, *f*(*K*_Ⅰ_, *K*_Ⅱ_) = 0.015630523 > 0, it can be judged that the mixed mode I and II cracks in the ice layer were unstable and expanded. In the area adjacent to the rim of the double wheel gap, the ice layer mainly shows the characteristics of crack radiation expansion dominated by shear. In this region, due to the existence of multiple initial defect cracks with random distribution of location and orientation, the composite crack forms a typical radial crack morphology during the unstable propagation process, which is consistent with the observation phenomenon of the indoor ice-breaking test. Under the repeated action of cyclic traffic load, when the crack propagation length reaches the critical size or the crack distribution density reaches the saturation degree, the interaction between the cracks will cause the stress concentration effect, which will eventually lead to local brittle fracture and the interfacial debonding of the ice layer. This mechanical process is the key mechanism for the rubber polyurethane gel pavement to realize an active ice-breaking function.

## 3. Conclusions

(1) The contact angle of rubber particles treated with NaOH, NaClO and KH550 decreased by 22.5%, 30.2% and 36.7%, respectively. NaOH solution improves the adhesive strength of rubber particles and polyurethane gels by eliminating zinc stearate on the rubber surface and forming a rough porous surface. The NaClO solution promoted the penetration of the adhesive molecules by removing the residual solvent on the surface of the rubber particles and forming a pore structure, thereby enhancing the bonding strength. The KH550 solution further improves the roughness of the surface of the rubber particles and the number of corrosion pores; enhances the air-entraining effect, fluidity and interfacial bonding strength; and significantly improves the working performance of the rubber particle mixture. After surface modification by three methods, the element types of rubber particles were significantly reduced, among which the element types of rubber particles treated by the KH550 solution decreased the most, indicating that the removal effect of surface impurities was the most significant.
(2) The freeze–thaw splitting strength of the mixture with four kinds of rubber particles increased greatly when the amount of polyurethane gel reached 10%. Under the condition of fixed rubber particle content, the growth trend of freeze–thaw splitting strength of the specimen increases first and then slows down with the increase in polyurethane gel content. When the content of rubber particles is 10%, the splitting strength ratio increases by 1.1%, 1.4%, 0.5% and 0.3%, respectively, when the content of polyurethane gels increases from 9–11%. When the content of polyurethane gels is 10%, its water damage resistance can meet the requirements of use, and the optimum polyurethane gel content of the mixture is determined to be 10%.
(3) With an increase in rubber particle content, the dynamic stability of the polyurethane gel mixture shows a trend of gradual increase, which is 6.2%, 18.2% and 23.5% higher than that of undoped rubber particles, respectively, and much higher than that of asphalt mixture, indicating that its anti-rutting performance is significantly enhanced. Compared with the mixture without rubber particles, the maximum bending strain increased by 11.8%, 25.1% and 31.7%, respectively, showing excellent deformation ability. The residual stability increased first and then decreased with the increase in rubber particle content, and the residual stability increased by 1.4%, 3.3% and 0.5%, respectively. The anti-scattering performance increased with the increase in rubber content, and an excessive amount would lead to an increase in scattering loss rate, but it could still be maintained below 5%, indicating that it still had good water stability under immersion conditions. Compared with the AC-13 asphalt mixture, the fatigue life of four kinds of rubber particle content mixtures increased by 2.9 times, 3.8 times, 4.3 times and 4.0 times, respectively. The fatigue performance of the polyurethane gel mixture decreased with an increase in rubber particle content. Compared with the ordinary asphalt mixture, the friction coefficient of four kinds of rubber particle content increased by 14.7%, 16.1%, 19.1% and 22.3%, respectively, which further verified its excellent skid resistance.
(4) Compared with the traditional asphalt mixture, RPGM shows superior de-icing performance, and with an increase in rubber particle content, its ice-breaking effect is significantly improved. Compared with the AC-13 asphalt mixture, the BPN change values of polyurethane gel mixtures with different rubber particle contents increased by 0.6 times, 1.8 times, 2.4 times and 2.8 times, respectively, under the rolling time of 30 min. The BPN change values of 5%, 10% and 15% RPGMs increased by 71.4%, 109.5% and 133.3%, respectively, compared with those of polyurethane gel mixtures without rubber particles. The average weighted recovery of the ice thickness to the pendulum value and the average weighted recovery rate of the pendulum value decrease with an increase in ice thickness, especially when the ice thickness is 1–6 mm. When the ice thickness reaches 9 mm, the two tend to be stable, indicating that the ice-breaking ability of the RPGM is significantly weakened. Under the action of traffic load, the RPGM can make the ice layer gradually fall off through stress, thermal effect and interface damage, indicating that RPGM exhibits strong potential for field application, subject to further long-term durability verification in real-world conditions. The ice layer damage is characterized by a mixed fracture mode dominated by a type II fracture.

## 4. Materials and Methods

### 4.1. Rubber Particles

In this paper, the cube-shaped rubber particles provided by Lingshou County Tairun Mineral Products Co., Ltd. (Shijiazhuang City, Hebei Province, China ) are selected, which are concave and convex, with no obvious agglomeration on the surface and burr phenomenon. The specific technical indicators are shown in Table 5, all of which meet the technical requirements of existing road rubber particles.

### 4.2. Polyurethane Gels

In this study, a two-component polyurethane gel provided by Jiangxi Zhanbang Technology Co., Ltd. (Fuzhou City, Jiangxi Province, China) was composed of polyols (component A) and isocyanates (component B). Referring to the relevant technical specifications [32,33,34], the performance of the selected two-component polyurethane gels were tested. The test results are shown in Table 6.

### 4.3. Modifier

(1) NaOH modifier

In this study, NaOH solution provided by Zibo Linnuo Chemical Co., Ltd. (Zibo City, Shandong Province, China) was used. NaOH solution shows a very broad application prospect in the engineering practice of rubber particle surface modification due to its simple modification process and low cost. In this study, NaOH was used as a granular solid, and the effective component content was 96%.

(2) NaClO modifier

In this study, sodium hypochlorite provided by Tianjin Tongxin Chemical Co., Ltd. (Tianjin, China) was used. The modification process of sodium hypochlorite is relatively simple. It is a common modification method to modify the surface of rubber particles by sodium hypochlorite. The index of NaClO solution used in this study is shown in Table 7.

(3) KH550 modifier

In this study, KH550 silane coupling modifier provided by Guangzhou Yinuo Chemical Technology Co., Ltd. (Guangzhou City, Guangdong Province, China ) was used. Silane coupling agent is widely used in the field of surface modification of rubber particles. In this study, the analytically pure KH550 silane coupling modifier was used, and the main physical properties are shown in Table 8.

### 4.4. Natural Aggregate

The natural aggregates used in the test are provided by Zhengzhou Zhengfa Municipal Construction Co., Ltd., Zhengzhou, China, and the technical indicators of the materials used are shown in Table 9.

### 4.5. Rubber Particle Performance Test

(1) Contact angle test

The German LAUDA Scientific (Lauda-Königshofen. Germany) contact angle measuring instrument LSA100 was used to microscopically analyze the surface of rubber particles with different treatment methods. The morphological characteristics of the gas–solid–liquid three-phase interface were captured by a high-resolution microscopic imaging system. The water contact angle of rubber particles after different modification treatments can accurately evaluate the improvement effect of surface wettability and provide a theoretical basis for optimizing the interface compatibility between rubber particles and cementitious material matrix.

(2) SEM test

Scanning electron microscopy (SEM) (provided by Shenyang Huayi Times Technology Co., Ltd. in Shenyang, Liaoning Province, China) can scan the surface morphology of the sample in a vacuum environment through a high-focusing electron beam and image it in combination with the principle of electron optics. It has the advantages of high resolution, continuous magnification and simple sample preparation. In this paper, the SEM test was used to observe the microstructure of the rubber particles, and the changes in the surface morphology of the steel slag before and after the optimization treatment were analyzed. The field emission scanning electron microscope with the model Quanta 450 was selected.

(3) EDS test

Energy-Dispersive Spectroscopy (EDS) is a micro-area composition analysis technology based on scanning electron microscopy. By detecting the energy distribution of these characteristic X-rays, the elemental composition and relative content of the sample surface can be qualitatively or quantitatively analyzed. Combined with the morphological changes from the scanning electron microscope, the changes in the surface elements of the original rubber particles and different modified rubber particles can be explored.

### 4.6. Gradation Design

**Figure 19:**
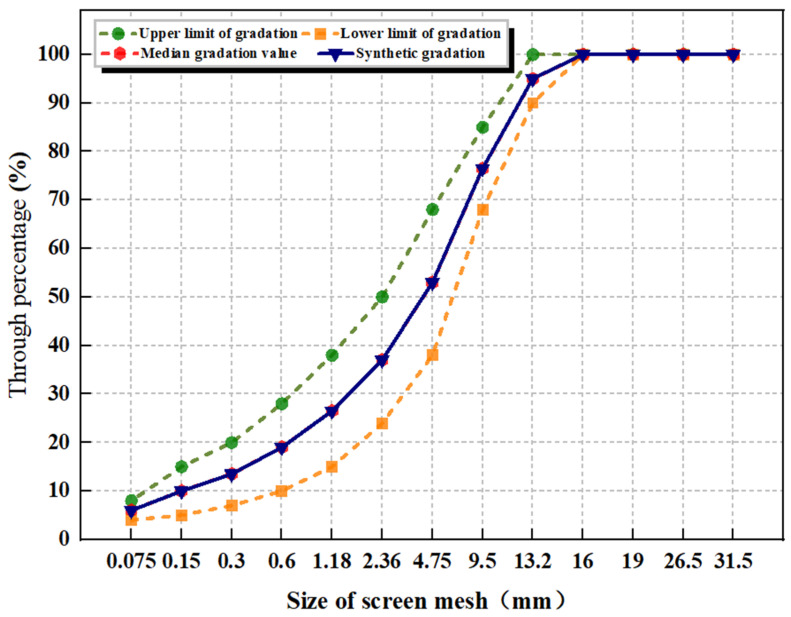
Synthetic grading curve.

With reference to the gradation design method of asphalt mixture, in order to take into account the structural strength and deformation performance of the mixture, this paper takes the AC-13 asphalt mixture gradation as the benchmark and uses the gradation median as the design gradation. The gradation curve is shown in Figure 19.

### 4.7. Determination of Polyurethane Gel Dosage

In order to determine the optimal amount of polyurethane gels, this paper selects the freeze–thaw splitting strength ratio as the evaluation index. The ratio of the splitting strength after freeze–thaw to the splitting strength before freeze–thaw is the splitting strength ratio. The freeze–thaw splitting strength of four kinds of rubber particles under different polyurethane gel dosages is measured. The splitting strength of the specimen is calculated according to Formulas (11) and (12).

(11) $$R_{T1}=0.006287\times P_{T1}/h_{1}$$

(12) $$R_{T2}=0.006287\times P_{T2}/h_{2}$$

where *R_T1_* is the splitting tensile strength of the first group of single specimens without freeze–thaw cycles, MPa; *R_T_*_2_ is the splitting tensile strength of the second group of single specimens subjected to freeze–thaw cycles, MPa; *P_T_*_1_ is the test load value of the first group of single specimens, N; *P_T2_* is the test load value of the second group of single specimens, N; *h*_1_ is the height of the first group of single specimens, mm; and *h*_2_ is the height of the second group of single specimens, mm.

### 4.8. Road Performance Test of Mixture

#### 4.8.1. High-Temperature Performance

The rutting test was used to evaluate the high-temperature stability of RPGM. The rutting tester model is HYCZ-5C, the test temperature is 60 °C, the load wheel pressure is 0.7 MPa, the deformation of the test time is 45 min and 60 min and the dynamic stability is calculated according to Formula (13).

(13) $$DS=\frac{\left(t_{2}-t_{1}\right)\times N}{d_{2}-d_{1}}\times C_{1}\times C_{2}$$

where *DS* is the dynamic stability of mixture, time/mm; *d*_1_ and *d*_2_ are the deformations at t_1_ (45min) and t_2_ (60min), mm; *C_1_* is the type correction coefficient of the testing machine, and the value is 1.0; *C_2_* is the coefficient of the specimen, and the value is 1.0; and N is the rolling speed of the test wheel, which is 42 times/min.

#### 4.8.2. Low-Temperature Performance

The low-temperature bending test was used to evaluate the low-temperature crack resistance of RPGM. The test temperature was −10 °C, and the constant rate of 50 mm/min was loaded until the specimen was destroyed. The failure strain was calculated according to Equation (14).

(14) $$\varepsilon_{B}=\frac{6\times h\times d}{L^{2}}$$

where ε_B_ is the maximum bending strain, *με; h* is the height of the specimen, mm; *L* is the span of the specimen, mm; and *d* is the mid-span deflection when the specimen is destroyed, mm.

#### 4.8.3. Water Stability

The water damage resistance of RPGM was evaluated by the immersion Marshall test and the immersion dispersion test. Two groups of standard Marshall specimens were prepared, with 4 in each group. One group was soaked in a 60 °C water bath for 30 min, and the stability was tested. The other group was soaked for 48 h, and the stability was tested. The stability of the two groups was tested, and the residual stability was calculated according to Formula (15).

(15) $$MS=\frac{MS_{1}}{MS_{0}}\times 100$$

where *MS* is the immersion residual stability of the specimen,%; *MS*_1_ is the stability of the specimen after soaking for 48 h, kN; and *MS*_0_ is the stability of the specimen after soaking for 30 min, kN.

The specimens after curing were divided into two groups: one group to carry out the standard dispersion test, and the other group was immersed in a water bath at a constant temperature of 60 °C for 48 h. After the immersion, the specimens were taken out and allowed to stand at room temperature for 24 h. Then, the scattering test was carried out on the specimens, and the mass loss of the specimens before and after immersion was measured. The scattering loss of the mixture was calculated according to Formula (16).

(16) $$\Delta S=\frac{m0-m1}{m0}\times 100$$

where ∆S is the scattering loss of asphalt mixture, %; $m_{0}$ is the mass of the specimen before the test, g; and $m_{1}$ is the residual mass of the specimen after the test, g.

#### 4.8.4. Fatigue Property

The fatigue performance of RPGM was verified by the trabecular three-point bending test. The rutting plate specimen after curing is divided into a 30 mm × 35 mm × 250 mm small beam specimen, and the force is applied to the position of the specimen at the two ends of the distance. The fatigue test is carried out under three stress ratios of 0.3, 0.4 and 0.5. The loading frequency is 10 Hz and the test temperature is 15 °C.

#### 4.8.5. Slip Resistance

According to the method of JTG 3450-2019 in ‘Highway Subgrade and Pavement Field Test Procedures’ [31], the pendulum friction coefficient tester is used to measure and record the test temperature and the measured pendulum value BPN.

(17) $${\mathrm{BPN}}_{20}={\mathrm{BPN}}_{T}+\Delta \mathrm{BPN}$$

where *BPN_20_* is converted to the pendulum value at standard temperature 20 °C, *BPN_T_* is the pendulum value of road surface temperature T, and *ΔBPN* is the temperature correction value.

### 4.9. Indoor Ice-Breaking Test

**Figure 20:**
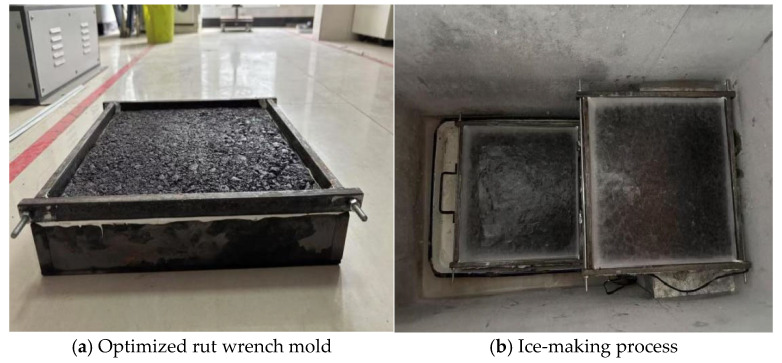
Ice-breaking test.

**Figure 21:**
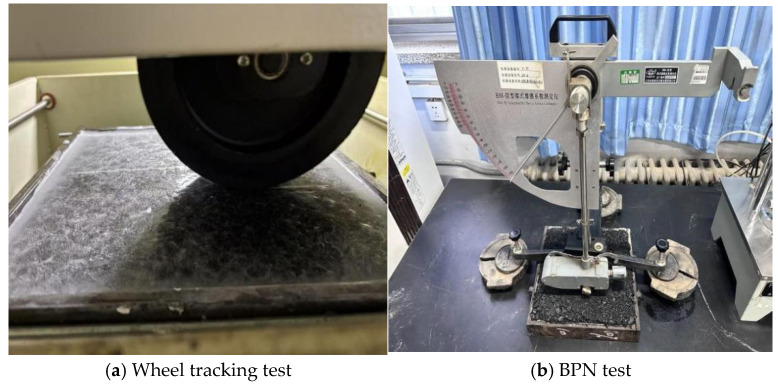
Ice-breaking test.

By simulating the actual traffic load mechanism, the rutting test can simulate the service environment of different working conditions by regulating multiple parameters, such as load, test temperature and geometric size. In view of these characteristics, the simulation method can be applied to the evaluation of pavement de-icing performance. In order to quantitatively characterize the ice-breaking effect of the road surface, this study uses the pendulum value test method, controls the ice thickness through the modified rutting plate mold and sets the rolling time to 50 min. We analyzed the pendulum value change value under different wheel rolling times and quantitatively evaluated the ice-breaking ability of the RPGM (as shown in Figure 20 and Figure 21).

## Figures and Tables

**Table 1:** Contact angle test results of rubber particles–water surface.

Surface Modification Methods	Without Modification	NaOH Modification	NaClO Modification	KH550 Modification
contact angle (°)	125.5	88.6	79.8	72.3

**Table 2:** Energy spectrum composition table of rubber particles with different modification methods.

Without Modification			NaOH Modification			NaClO Modification			KH550 Modification		
ele.	Wt%	At%	ele.	Wt%	At%	ele.	Wt%	At%	ele.	Wt%	At%
C	59.3	69.7	C	67.2	74.9	C	60.1	70.2	C	61.4	70.7
O	17.2	18.8	O	21.9	21.4	O	26.3	25.3	O	21.1	20.8
Zn	17.9	6.7	Zn	6.3	1.4	Zn	9.2	2.1	Zn	7.6	2.2
Cl	1.5	2.6	Cl	1.9	1.0	Cl	3.5	1.7	Cl	1.8	1.7
Zr	0.6	0.3	Zr	0.2	0.2	Zr	0.3	0.1	Si	4.3	2.8
S	0.2	0.3	S	1.1	0.4	S	0.2	0.4	Ca	0.2	0.1
K	0.3	0.1	Ca	1.2	0.4	Al	0.1	0.1	N	3.6	1.7
Ca	2.5	0.4	Al	0.2	0.3	Ca	0.3	0.1	—		
Al	0.5	1.1	—			—			—		

**Table 3:** Temperature correction value.

Temperature (°C)	0	5	10	15	20	25	30	40
temperature correctionΔBPN	−6	−4	−3	0	+2	+3	+5	+7

**Table 4:** BPN test results.

Mix Type	BPN Value			Mean Value	Standard Deviation	Coefficient of Variation
	1	2	3			
AC-13 Asphalt mixture	71.2	70.4	69.4	70.3	0.90	1.28
0% RPGM	79.4	81.8	80.5	80.6	1.20	1.49
5% RPGM	81.6	82.3	80.9	81.6	0.70	0.86
10% RPGM	83.4	84.9	82.7	83.7	1.12	1.34
15% RPGM	85.9	85.4	86.8	86.0	0.71	0.82

**Table 5:** Technical index of rubber particles.

Technology Index	Moisture Content/%	Apparent Density/g/cm^3^	Shore Hardness/%	Elastic Modulus/MPA	Slender Flat Particle Content/%	Carbon Black Content/%
technical requirement	<1	1.10–1.30	55–80	≥5	≤10	25–38
test result	0.22	1.153	69	10	3.9	32

**Table 6:** Properties of polyurethane gel binder.

Testing Item	Technical Requirement	Test Result
density	actual measurement	1.4 g/cm^3^
viscosity	≥1	3.5 mPa·s
tensile strength	≥2	7.0 MPa
breaking elongation	≥150	298%
surface dry time	-	2 h
hard drying time	-	10 h

**Table 7:** Technical index of sodium hypochlorite solution.

Project	Purity	Active Chlorine	Free Alkali (in NaOH)	Fe	As	Heavy Metals (in Pb)
index	analytically pure	≥5.5%	0.1–1.0%	≤0.003%	≤0.0005%	≤0.002%

**Table 8:** Main physical properties of KH550.

Physical Property	Appearance	Boiling Point/°C	Specific Gravity	Solubility
Numerical and Description	Colorless Liquid	208	0.947	Soluble in water and organic solvents

**Table 9:** Technical indexes of natural aggregate.

NO.	Type	Materials	Technical Indexes
1	Coarse aggregate	Basalt	Apparent specific gravity (13.2 mm~16 mm) 2.745 Apparent specific gravity (9.5 mm~13.2 mm) 2.736 Apparent specific gravity (4.75 mm~9.5 mm) 2.724 Apparent specific gravity (2.36 mm~4.75 mm) 2.753 Water absorption (13.2 mm~16 mm) 1.1% Water absorption (9.5 mm~13.2 mm) 0.9% Water absorption (4.75 mm~9.5 mm) 0.7% Water absorption (2.36 mm~4.75 mm) 1.2% Crush value 18.2%
2	Fine aggregate	Limestone	Apparent specific gravity (1.18 mm~2.36 mm) 2.776 Apparent specific gravity (0.6 mm~1.18 mm) 2.792 Sand equivalent (1.18 mm~2.36 mm) 75% Sand equivalent (0.6 mm~1.18 mm) 77%
3	Mineral fines	Limestone	Apparent specific gravity 2.654 No agglomeration Hydrophilicity 0.72

## Data Availability

Some or all of the data, models, or code that support the findings of this study are available from the corresponding author upon reasonable request.

## References

[1] Liu T., Guo N., Tan Y., You Z., Jin X. (2020). Research status and development trend of phase change materials for road use. Mater. Introd..

[2] Das B.P., Das S., Siddagangaiah A.K. (2021). Probabilistic modeling of fatigue damage in asphalt mixture. Constr. Build. Mater..

[3] Tan Y., Li J., Xu H. (2020). Perception and early warning of snow and ice conditions on cold roads. Basic Sci. China.

[4] Dong Q., Du Y.C., Guo M. (2024). Review of Academic Research on Pavement Engineering in China. Chin. J. Highw..

[5] Liu Z., Sha A., Jiang W. (2019). Salt asphalt pavement research progress: Salt material, mixture and its performance and evaluation. Chin. J. Highw..

[6] Sajid H.U., Kiran R., Qi X., Bajwa D.S., Battocchi D. (2020). Employing corn derived products to reduce the corrosivity of pavement deicing materials. Constr. Build. Mater..

[7] Chen Y., Tan Y., She H., Zhang M., Jiang X., Guo P., Li Y., Zhang Y. (2023). Characteristics of slow-release ice and snow melting micro-surfacing materials. Int. J. Pavement Eng..

[8] Chen Y., Li Z., Zhao C., Guo T., Wang Z., Li J., Gao D. (2020). Study on environmentally friendly slow-release active melting ice and snow coating materials. Chin. J. Highw..

[9] Li Z., Guo T., Chen Y., Yu L., Niu X., Yang X., Jin L. (2022). Study on road performance and electrothermal performance of poured conductive asphalt concrete. Adv. Mater. Sci. Eng..

[10] Wang C., Han X., Chen J., Hou R., Zheng S. (2018). Conductive heat effect of guss conductive asphalt concrete. Mater. Introd..

[11] Hasan R., Ali A., Decarlo C., Elshaer M., Mehta Y. (2021). Laboratory evaluation of electrically conductive asphalt mixtures for snow and ice removal applications. Transp. Res. Rec..

[12] Tan Y., Zhang C., Xu H., Tian D. (2019). Summarization of research on snow melting and ice melting characteristics and road performance of active de-icing pavement. Chin. J. Highw..

[13] Chen Y., Li Z. (2013). Deicing mechanism of crumb rubber asphalt pavement. J. Cent. South Univ. (Nat. Sci. Ed.).

[14] Liang C., Zhang H., Gu Z., Xu X., Hao J. (2020). Study on Mechanical and Viscoelastic Properties of Asphalt Mixture Modified by Diatomite and Crumb Rubber Particles. Appl. Sci..

[15] Li S., Ke Y., Xie L., Zhao Z., Huang X., Wang Y., Wang Z. (2023). Study on the aging of three typical rubber materials under high-and low-temperature cyclic environment. e-Polymers.

[16] Ma Z., Wei H., Wei D., Jiang B., Wang X. (2025). Study on snow melting performance evaluation and optimization design of conductive rubber electric heating pavement. Constr. Build. Mater..

[17] Shan W., Zhang S. (2025). Study on the road performance of terminal carboxylated nitrile rubber-modified epoxy asphalt permeable concrete. Materials.

[18] Sun M., Hou D., Geng L., Yan Z., Bi Y., Huang Z., Ren S., Wang B. (2024). Study on road performance and mechanical properties of multi-gravel polyurethane concrete. Silic. Bull..

[19] Cao Z., Hao Q., Xu S., Han X., Yi J., Sun G. (2025). Preparation and performance evaluation of bio-based polyurethane modified asphalt binders: Towards greener and more sustainable asphalt modifier. Constr. Build. Mater..

[20] Hesami S., Sadeghi V., Azizi A. (2019). Investigation of modified bitumen’s rheological properties with synthesized polyurethane by MDI-PPG reactive prepolymers. J. Thermoplast. Compos. Mater..

[21] Torzs T., Lu G., Monteiro A.O. (2019). Hydraulic properties of polyurethane-bound permeable pavement materials considering unsaturated flow. Constr. Build. Mater..

[22] Zheng Y., Han S., Zheng H. (2025). Design, fabrication, and laboratory performance evaluation of polyurethane poroelastic road surfaces (PERS) mixtures. Constr. Build. Mater..

[23] Gao J., Chen J., Meng X., Wang H., Xu N. (2023). Research on the selection of polyurethane adhesive and direct tensile properties of polyurethane rubber particle mixture. Case Stud. Constr. Mater..

[24] Chen J., Xie M., Hao W., Xie P., Huang W. (2018). Experimental study on anti-icing and deicing performance of polyurethane concrete as road surface layer. Constr. Build. Mater..

[25] Chen J., Yin X., Wang H., Ding Y. (2018). Evaluation of durability and functional performance of porous polyurethane mixture in porous pavement. J. Clean. Prod..

[26] Zou B., Yin J., Cao C., Long X. (2025). Mechanical performance analysis of rubber elastic polymer-polyurethane reinforced cement-based composite grouting materials. J. Polym. Mat..

[27] Agaavriloaie L., Oprea S., Barbuta M., Florentina L. (2012). Characterisation of polymer concrete with epoxy polyurethane gel acryl matrix. Constr. Build. Mater..

[28] Min S., Bi Y., Zheng M., Chen S., Li J. (2019). Evaluation of a cold-mixed high-performance polyurethane mixture. Adv. Mater. Sci. Eng..

[29] Li G., Tian X., Gao K., Wu Q., Huang S., Xie Z. (2024). Study on the performance and microscopic mechanism of activated crumb rubber synergistic polyurethane composite modified asphalt. Constr. Build. Mater..

[30] Wang D., Liu P., Leng Z., Leng C., Lu G., Buch M., Oeser M. (2017). Suitability of PoroElastic Road Surface (PERS) for urban roads in cold regions: Mechanical and functional performance assessment. J. Clean. Prod..

[31] (2019). Specification for Field Test of Highway Subgrade and Pavement.

[32] (2008). Test Method for Architectural Waterproofing Coatings.

[33] (2011). Determination of Relative Density of Chemical Products.

[34] (2011). Test code for asphalt and asphalt mixture of highway engineering.

